# Identification and Verification on Prognostic Index of Lower-Grade Glioma Immune-Related LncRNAs

**DOI:** 10.3389/fonc.2020.578809

**Published:** 2020-11-23

**Authors:** Jing Wen, Youjun Wang, Lili Luo, Lu Peng, Caixia Chen, Jian Guo, Yunlong Ge, Wenjun Li, Xin Jin

**Affiliations:** ^1^ Xiamen Key Laboratory of Chiral Drugs, School of Medicine, Xiamen University, Xiamen, China; ^2^ Department of Neurosurgery, Xiang’an Hospital of Xiamen University, Xiamen University, Xiamen, China

**Keywords:** lower-grade glioma, lncRNA, immune, prognosis model, enrichment analysis of gene function

## Abstract

Previous studies have shown that the prognosis of patients with lower-grade glioma (LGG) is closely related to the infiltration of immune cells and the expression of long non-coding RNAs (lncRNAs). In this paper, we applied single-sample gene set enrichment analysis (ssGSEA) algorithm to evaluate the expression level of immune genes from tumor tissues in The Cancer Genome Atlas (TCGA) database, and divided patients into the high immune group and the low immune group, which were separately analyzed for differential expression. Venn analysis was taken to select 36 immune-related lncRNAs. To construct a prognostic model of LGG based on immune-related lncRNAs, we divided patients into a training set and a verification set at a ratio of 2:1. Univariate Cox regression and the Least Absolute Shrinkage and Selection Operator (LASSO) regression were performed to select 11 immune-related lncRNAs associated with the prognosis of LGG, and based on these selected lncRNAs, the risk scoring model was constructed. Through Kaplan-Meier analysis, the overall survival (OS) of patients in the high-risk group was significantly lower than that of the low-risk group. Then, established a nomogram including age, gender, neoplasm histologic grade, and risk score. Meanwhile, the predictive performance of the model was evaluated by calculating the C-index, drawing the calibration chart, the clinical decision curve as well as the Receiver Operating Characteristic (ROC) curve. Similar results were obtained by utilizing the validation data to verify the above consequences. Based on the TIMER database, the correlation analysis showed that the 11 immune-related lncRNAs risk score of LGG were in connection with the infiltration of the subtypes of immune cells. Subsequently, we performed enrichment analysis, whose results showed that these immune-related lncRNAs played important roles in the progress of LGG. In conclusion, these 11 immune-related lncRNAs have the potential to predict the prognosis of patients with LGG, which may play a key role in the development of LGG.

## Introduction

Glioma is the most common type of primary tumors in the nervous system (1, 2). Previous studies have shown that the immune infiltration microenvironment of lower-grade glioma (LGG) was strongly linked with the prognosis of patients (3). Lu et al. screened three tumor infiltrated immune cell subsets associated with malignant transformation of LGG and constructed a prediction model, which aimed at transforming LGG to a higher grade based on immune-related characteristics (4). There was a strong correlation between the increased survival in LGG and complementarity of the IDH1 mutant to the CDR3 domain of the T-cell receptor beta chain (5). Given that the prognosis of patients with LGG tumor was closely related to the immune microenvironment, furthermore, in order to improve the prognosis of LGG and provide theoretical support for the study of its immunotherapy, we screened reliable immune-related prognostic factors to construct a clinical prognosis model of LGG, and explored the potential functions of these immune-related factors through enrichment analysis.

lncRNA is a non-coding RNA with a length of more than 200 nucleotides. A large number of lncRNAs have been found to regulate gene expression through various pathways and participate in the progression of various tumors throughout the body (6, 7). LncRNAs have important effects on the immunity, proliferation, migration, apoptosis, and autophagy of the tumor. Zhao et al. found that lncRNA MEG3 could regulate the proliferation, apoptosis and autophagy of gliomas, which affected the prognosis of patients (8). The expression of lncRNA GAS5 in patients with LGG was related to prognosis, whose potential function included the regulation of ribosomal biogenesis and translation (9). Previous studies have shown that lncRNA as a tumor prognostic marker had broad prospects in the prognosis of LGG.

Immune-related lncRNAs have uniquely potential values in the prognosis of patients with gliomas. For example, overexpression of lncRNA HOTAIRM1 was associated with the immune activation in glioma tissues, whose character lied in enhancing T cell-mediated response and inflammatory response. lncRNA HOTAIRM1 could be used as the prognostic biomarker for glioma patients and a potential target for the treatment of gliomas (10, 11). Song et al. discovered four types of lncRNAs related to the pathological grade and prognosis of patients with gliomas (12). lncRNAs have been verified as a prognostic marker in many cancers. Sun et al. identified that lncRNAs were related to tumor immune infiltration, and they constructed a computational model so as to improve the prognosis and immunotherapy response of patients with non-small-cell lung cancer (13). Shen et al. confirmed 11 immune-related lncRNAs based on the single-sample gene set enrichment analysis (ssGSEA) to construct a prognostic model for breast cancer patients, which performed well in the prediction (14). However, there is rarely research performed by ssGSEA to construct a prognostic model based on identifying immune-related lncRNAs in LGG.

In this paper, we analyzed the data set of lncRNA expression in The Cancer Genome Atlas (TCGA) and screened for immune-related lncRNAs by ssGSEA. Based on the screened immune-related lncRNAs, the clinical prognostic model of LGG was constructed, and the potential mechanisms of immune-related lncRNAs of LGG were explored as well.

## Materials and Methods

### Technology Roadmap

**Figure 1:**
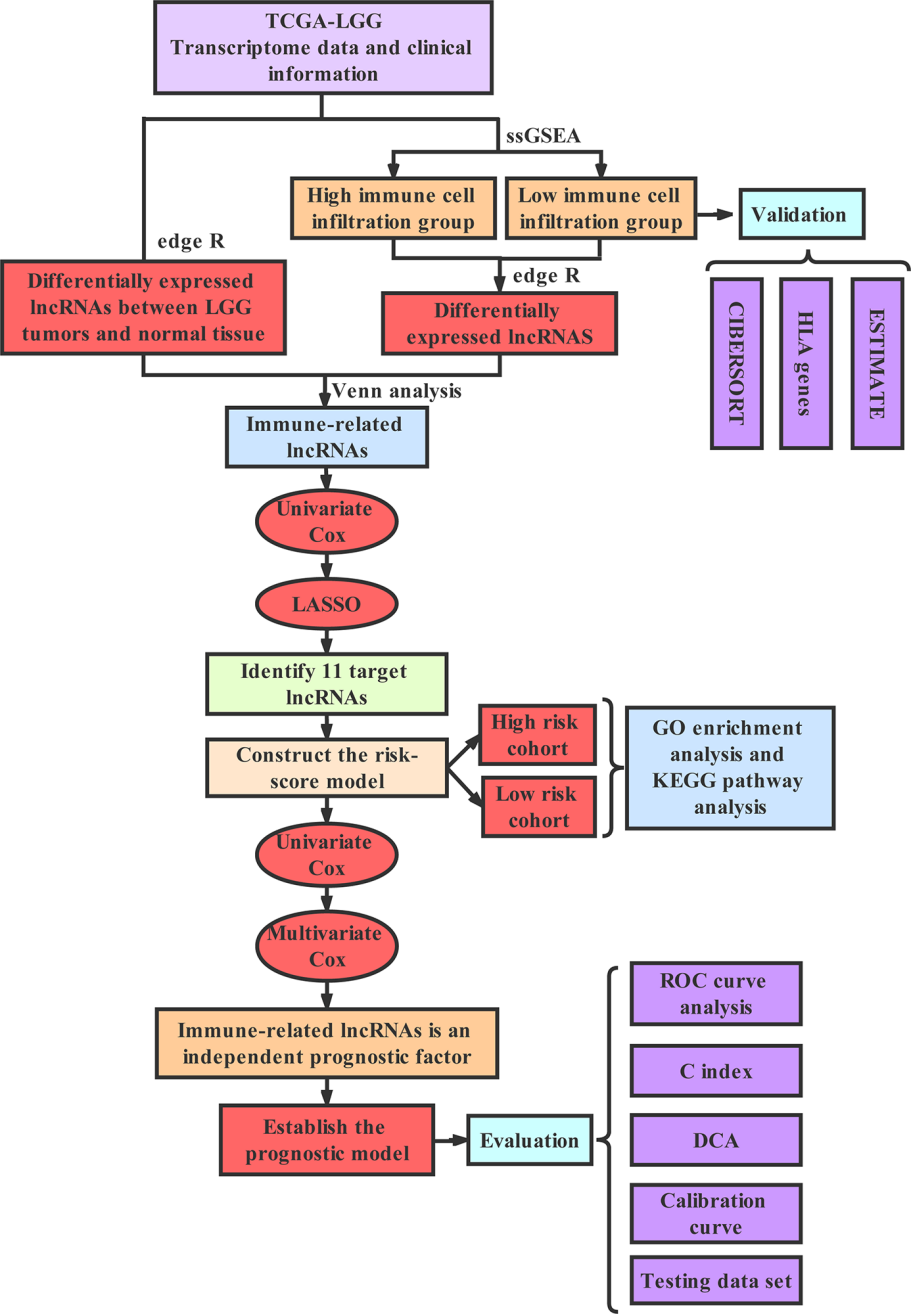
The technology roadmap.

The full text was analyzed according to the following technical procedure. The data were downloaded from TCGA and the patients were divided into the high immune group and the low immune group by ssGSEA. Immune-related lncRNAs were obtained by Venn analysis. Prognostic lncRNAs were further screened by Cox regression analysis and LASSO regression analysis. A risk scoring model and nomogram were constructed. Finally, ROC curve, C index and DCA were used to evaluate the model. The validation set was applied to validate all the results (**Figure 1**).

### Collecting and Grouping of Data

Both sequencing data and corresponding clinical data of LGG were selected from TCGA database (https://portal.gdc.cancer.gov). Based on the research of Bindea et al. (15), we analyzed 29 immune-associated gene sets (**Supplementary Material File 1**) which represented diverse immune cell types, functions, and pathways. We used the ssGSEA to quantify the activity or enrichment levels of immune cells, functions, or pathways in the LGG tissues. According to the results of ssGSEA, the LGG samples in TCGA were divided into high immune group and low immune group by “hclust” in RStudio. According to the research of Yoshihara et al. (16), the stromal score and the immune score as well as tumor purity of high immune group and low immune group were evaluated by Estimation of Stromal and Immune cells in Malignant Tumor tissues using Expression data (ESTIMATE) to verify the effect of ssGSEA grouping. In addition, we verified the effect of ssGSEA grouping by analyzing the expression level of human leukocyte antigen (HLA) between high and low immune groups. As Newman et al. believed (17), Estimating Relative Subsets of RNA Transcripts (CIBERSORT) algorithm could be used to determine the infiltration of various immune cells in the tumor samples. While, by comparing the infiltration level of various immune cells in the high immune group and the low immune group, the grouping effect of ssGSEA was verified again.

### Identification of Immune-Related lncRNA in LGG

Based on the ssGSEA analysis results above, the high and low immune groups were obtained by applying the undifferentiated cluster analysis. And the differential expression of lncRNA in the high immune group and the low immune group was analyzed by utilizing “edgeR” package with the criteria of | log_2_FC | > 0.5 and False Discovery Rates (FDR) < 0.05. The differential expression of lncRNAs between cancer and adjacent normal tissue were determined by the same method. In order to screen out the immune-related lncRNA, Venn analysis was undertaken to take the intersection of the above two difference analysis to obtain the immune-related lncRNAs.

### Identification of Immune-Related lncRNAs Associated With the Prognosis of LGG and the Construction of a Risk Scoring Model

Based on the clinical data of LGG in TCGA, patients with a follow-up time of more than 30 days were extracted, and all samples were used as the training set and the validation set of the model at a ratio of 2:1. We applied the training set to build a prediction model and used the validation set to verify the model. Based on the training set, the Univariate Cox regression and LASSO regression analysis were used to screen for immune-related lncRNA associated with the prognosis of patients with the standard of *p* < 0.05. Immune-related lncRNAs associated prognosis were subjected to Multivariate Cox regression to calculate their coefficients (coefi) respectively. Then, coefi and lncRNA expression levels (expi) were used to build a risk scoring model:$\text{Risk}\;\text{score}=\Sigma_{i=1}^{n}\;(coefi*expi)$. Taking the median risk score as the cut-off point so as to divide patients with LGG into high-risk group and low-risk group. The Kaplan-Meier survival curve was drawn aimed to analyzing whether the prognosis of the two groups was of great difference or not. Both Univariate Cox regression and Multivariate Cox regression analysis were used to assess the relationship between risk score, age, gender, tumor pathological grade and the prognosis of patients with LGG. The Receiver Operating Characteristic (ROC) curve was drawn to evaluate the predictive effectiveness of the risk scoring model. At last, the results above were verified with the validation set.

### The Construction of Nomogram and the Evaluation of Predicting the OS of Patients

We incorporated risk scores and clinical indicators into the model to optimize its predictive power. According to the results of Multivariate Cox regression, a nomogram was drawn. By drawing a calibration chart, the ROC curve was executed to obtain the area under the curve (AUC). The C-index was calculated and the clinical decision curve analysis (DCA) was performed to evaluate the predictive effect of the nomogram. Then, the validation set was used to verify the above results.

### Correlation Analysis of Immune Cell Infiltration

TIMER (https://cistrome.shinyapps.io/) is a database for detecting the infiltration of immune cells in tumor tissues by using RNA-Seq expression profile data. Infiltration data of B cells, CD4^+^T cells, CD8 ^+^T cells, dendritic cells, macrophages, and neutrophils can be downloaded from TIMER database. The correlation between risk scores and immune infiltration was calculated by Pearson correlation analysis.

### Gene Set Enrichment Analysis

We downloaded and installed GSEA software from the official website, including the KEGG gene collection file (C2.all.v6.2.symbols.gmt) and the GO functional gene collection file (C5.all.v6.2.symbols.gmt). We performed GO and KEGG enrichment analysis on the differentially expressed genes of the high-risk and low-risk groups, and deduced their functions by analyzing the gene set. In this paper, we explored whether differentially expressed genes between these two groups were enriched among immune-related biological functions or pathways.

## Statistical Analysis

All statistical analysis was accomplished by R version 3.6.2 (Institute for Statistics and Mathematics, Vienna, Austria; https://www.r-project.org) (Package: limma, GSVA, GSEABase, sparcl, pheatmap, estimate, ggpubr, e1071, preprocessCore, survival, glmnet, survminer, survivalROC, rms, foreign, timeROC, and ggplot2). The correlation was determined by Pearson correlation analysis. Chi-square test and t-test were utilized to compare clinical variables. Survival status was assessed by the Cox regression analysis. OS was generated by the Kaplan-Meier method and evaluated by the log-rank test. Two-tailed p < 0.05 was considered statistically significant.

## Results

### Grouping and Identification of LGG Samples

**Figure 2:**
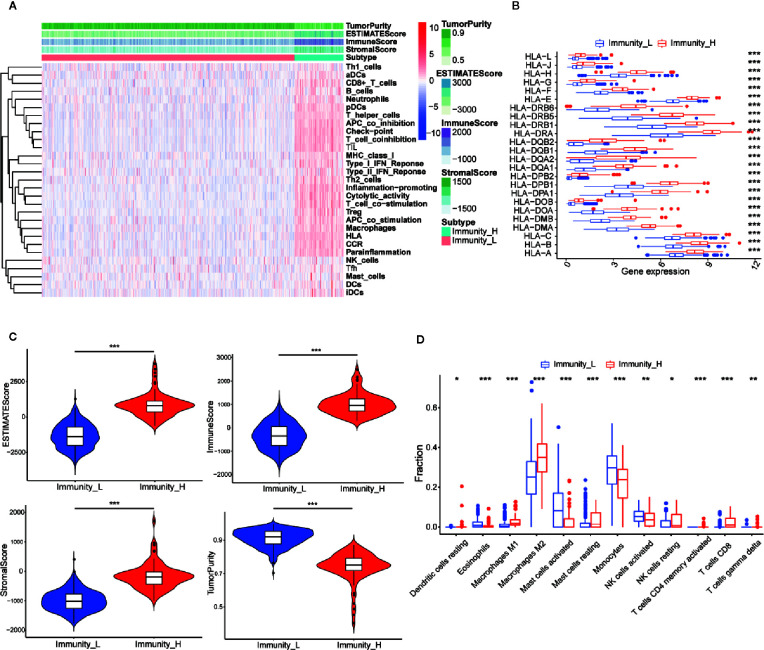
Establishment and verification of LGG grouping. **(A)** The heatmap showed the unsupervised clustering of 29 immune-associated gene sets in the high immune group (Immunity_H) and the low immune group (Immunity_L). Parameters including tumor purity, ESTIMATE scores, immune scores, and stromal scores. **(B)** The histogram showed the differences in expression levels of HLA and family gene between the high and low immunity groups. **(C)** The violin diagram showed the difference in tumor purity, ESTIMATE scores, immune scores and stromal scores. **(D)** The CIBERSORT algorithm obtained that the infiltration level of most immune-related cells in the high immune group is lower than that in the low immune group, such as Dendritic cells resting, Macrophages M1, Macrophages M2, Mast cells resting, NK cells resting, T cells CD4 memory activated, T cells CD8, and T cells gamma delta. *p < 0.05; **p < 0.01; ***p < 0.001.

The 529 LGG HTSeq-FPKM date was obtained from TCGA. The transcriptome of LGG samples was analyzed by ssGSEA algorithm to evaluate the expression level of immune gene set in tumor tissues of each patient. The unsupervised hierarchical clustering algorithm was performed to divide all patients into a high immune group (Immunity-H) (n = 86) and a low immune group (Immunity-L) (n = 443) (**Figure 2A**). At the same time, we found that the expression of HLA family genes in the high immune group was higher than that in the low immune group (**Figure 2B**). The violin chart showed that the ESTIMATE score, the immune score and the stromal score of the high immune group (Immunity-H) were lower than that of the low immune group, while the tumor purity of the low immune group was higher than that of the high immune group (**Figure 2C**). In addition, we found that most immune cells had higher abundance in the high immune group, such as Dendritic cells resting, Macrophages M1, Macrophages M2, Mast cells resting, NK cells resting, T cells CD4 memory activated, T cells CD8, and T cells gamma delta (**Figure 2D**). In conclusion, these results indicated that the grouping scheme of LGG was feasible and reliable.

### Screening and Identification of Immune-Related lncRNAs in LGG

**Figure 3:**
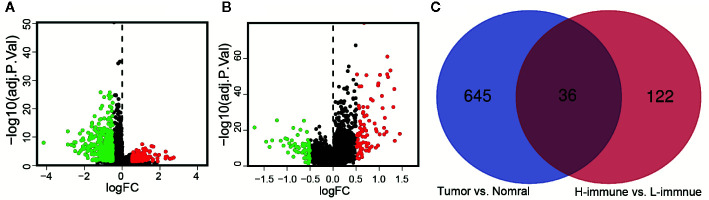
Identification of immune-related lncRNAs in LGG. **(A)** The volcano graph showed that 455 lncRNAs were down-regulated and that 226 lncRNAs were up-regulated in tissues of LGG compared to normal tissues. **(B)** The volcano graph showed that compared with the low infiltration group, 71 lncRNAs were down-regulated and that 87 lncRNAs were up-regulated in the high infiltration group. **(C)** Venn diagram showed that 36 lncRNAs behaved differently between tumor and normal tissues are differentially expressed between the low immune group and the high immune group.

According to the criteria of | log_2_FC | > 0.5 and FDR < 0.05, there were 681 differentially expressed lncRNAs between cancer and adjacent normal tissue (**Figure 3A**). The same method was used to obtain 158 differentially expressed lncRNAs between high and low immune groups (**Figure 3B**). After two-way Venn analysis, compared with the adjacent normal tissue and low immune group, 36 differentially expressed lncRNAs were detected in the tumor group and high immune group (**Figure 3C**) (**Supplementary Material File 2**). Based on the above methods, we finally screened out 36 immune-related lncRNAs in LGG.

### Identification of Prognostic Markers in 11 Immune-Related lncRNAs of LGG

**Figure 4:**
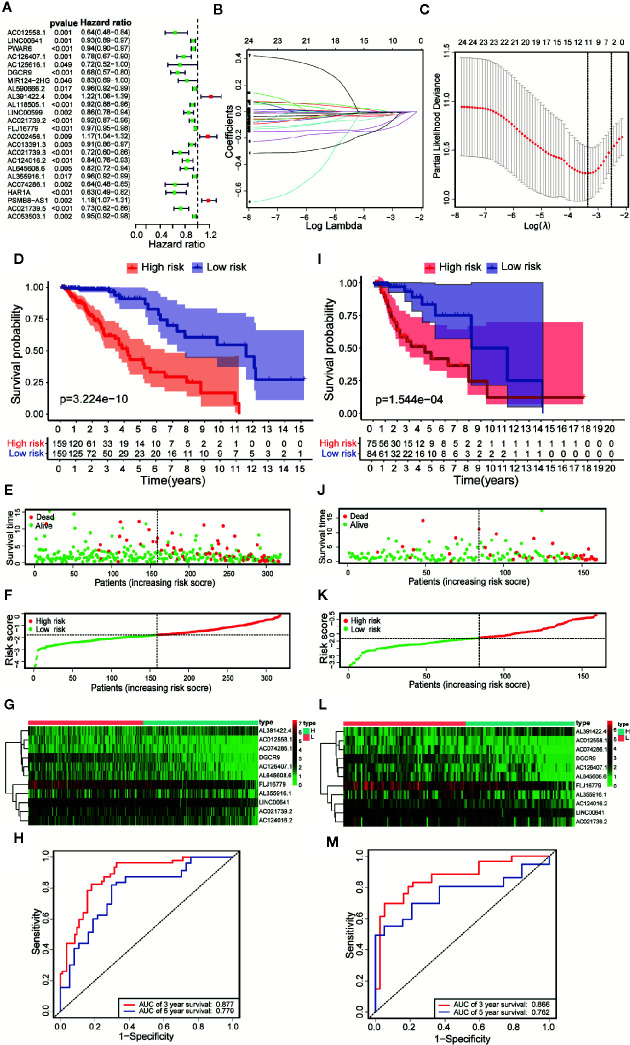
Constructing a risk scoring prognostic model of LGG based on immune-related lncRNAs. **(A)** Based on the Univariate COX regression analysis, the forest map showed that there are 24 immune-related lncRNAs with prognostic significance (*p* < 0.05). **(B)** LASSO regression analysis further screened out 11 lncRNAs that were closely related to prognosis. **(C)** Illustration of LASSO coefficient spectrum for prognosis-related lncRNAs. **(D)** Kaplan-Meier survival analysis showed that in the training data sets, patients in high-risk group have worse OS compared with those in low-risk group (*p* =3.224e-10). **(E)** Overview of the survival time for each patient in the training data sets. **(F)** The distribution of a risk score for each patient in training data sets. **(G)** Heatmaps of expression profiles for 11 lncRNAs between low-risk group and high-risk group in the training data sets. Warm colors represented high expression, while cold colors represented low expression. **(H)** ROC curve analysis of the 11 lncRNAs signature demonstrated that AUCs of 3-year and 5-year OS were 0.877 and 0.779 in the training data set. **(I)** Kaplan-Meier survival analysis showed that in the testing data sets, patients in high-risk group have worse OS compared with those in low-risk group (*p* =1.544e-04). **(J)** Overview of the survival time for each patient in the testing data sets. **(K)** The distribution of a risk score for each patient in testing data sets. **(L)** Heatmaps of expression profiles for 11 lncRNAs between low-risk group and high-risk group in the testing data sets. Warm colors represented high expression, while cold colors represented low expression. **(M)** ROC curve analysis of the 11 lncRNAs signature demonstrated that AUCs of 3-year and 5-year OS were 0.866 and 0.762 in the testing data set.

According to the follow-up data of patients with LGG in the training set, we applied Univariate Cox regression to determine a total of 24 prognostic-related lncRNAs on the criterion of *p* < 0.05 (**Figure 4A**). In order to reduce the overfitting among prognostic markers, LASSO regression was used to further choose 11 immune-related lncRNAs associated with prognosis (**Figures 4B, C** **)**.

### Construction and Evaluation of 11 Immune-Related lncRNAs Risk Score Prognostic Model for LGG

Based on Multivariate COX regression, these 11 immune-related lncRNAs were used to construct the risk scoring model $\left(\text{Risk}\;\text{score}=\Sigma_{i=1}^{11}(coefi*Expi)\right)$. Kaplan-Meier curve showed that the OS of the samples in the high-risk group was lower than that in the low-risk group (*p* = 3.224e-10, **Figure 4D**). The risk curve and the scatterplot showed the risk scores and survival status of each LGG sample (**Figure 4E**). The risk scores and mortality rate of the samples in the high-risk group were higher than those in the low-risk group (**Figure 4F**). The heatmap of 11 immune-related lncRNAs in the LGG samples showed their expression in both groups (**Figure 4G**). Aiming at evaluating the sensitivity and specificity of the risk scores for the prognosis of patients with LGG, a time-dependent ROC analysis was performed. The results of ROC curve analysis showed that AUCs of 3-year and 5-year OS were 0.877 and 0.779 respectively (**Figure 4H**). In summary, 11 immune-related lncRNAs were identified as prognostic markers for LGG, the risk scoring model based on immune-related lncRNAs was effective on prediction. What’s more, similar results were obtained by using the same methods on the validation set (**Figures 4I–M**).

### Evaluation of 11 Immune-Related lncRNAs Risk Score as Independent Prognostic Factors in Patients With LGG

**Figure 5:**
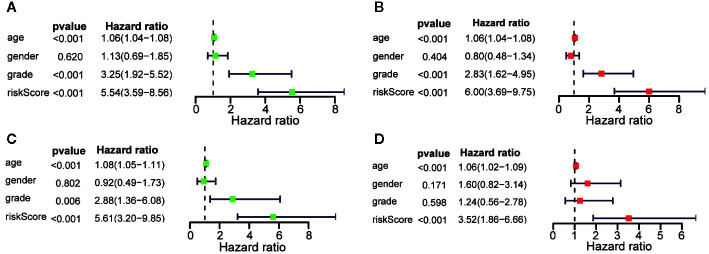
Assessment of the risk scores and the prognostic value of clinical variables. **(A)** Univariate Cox analysis showed that risk scores and clinical variables including age, and that neoplasm histologic grade were significantly related to OS in the training data sets. **(B)** Multivariate Cox analysis manifested that the 11 lncRNAs signature was an independent prognostic indicator for LGG in training data sets. **(C)** Univariate Cox analysis showed that risk scores and age were significantly related to OS in the testing data sets. **(D)** Multivariate Cox analysis manifested that the 11 lncRNAs signature was an independent prognostic indicator for LGG in testing data sets.

Univariate and Multivariate Cox regression were performed to analyze whether the above 11 immune-related lncRNAs were independent prognostic factors of clinical pathological factors (age, gender, and neoplasm histologic grade). The results showed that the risk scores of Univariate Cox regression analysis and 95% CI hazard ratio (HR) were 5.545 and 3.589-8.564 (p < 0.001), while the risk scores of Multivariate Cox regression analysis and 95% CI hazard ratio (HR) were 5.997 and 3.691–9.746 (p < 0.001), which indicated that 11 lncRNAs were independent prognostic factors for patients with LGG (**Figures 5A, B** **)**. Overall, these results indicated that 11 immune-related lncRNAs are independent prognostic factors for patients with LGG. Similar results were obtained in the validation set (**Figures 5C, D** **)**.

### Establishment of the Prognostic Nomogram and Evaluation of Predictive Effectiveness

**Figure 6:**
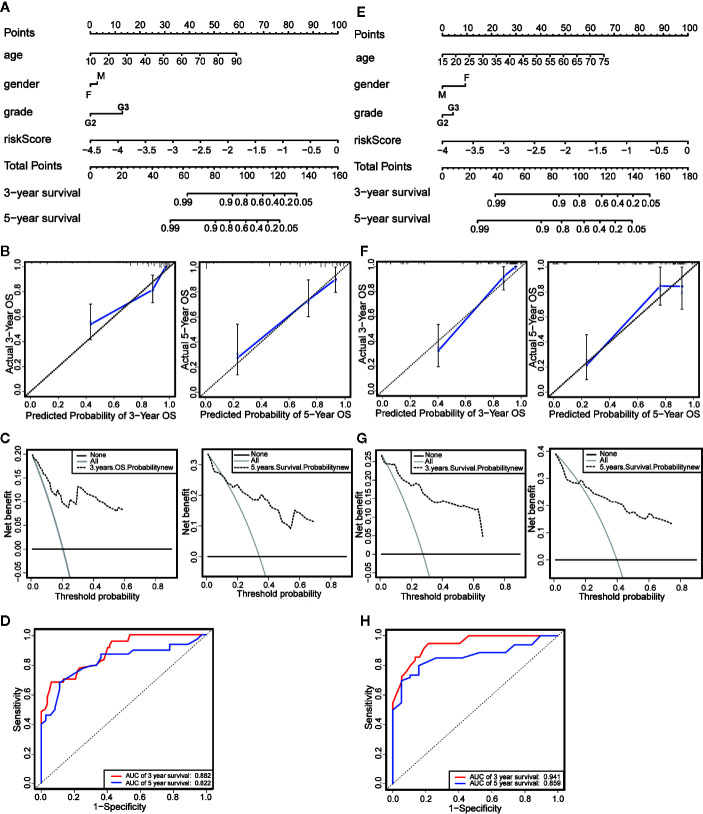
Establishment and evaluation of nomograms. **(A)** The nomograms for predicting the patients’ OS in the training data sets. **(B)** The calibration curve for 3-year OS and 5-year OS of the nomogram in the training data sets. **(C)** The DCA for 3-year OS and 5-year OS of the nomogram in the training data sets. **(D)** ROC curve analysis showed that AUCs of 3-year and 5-year OS were 0.882 and 0.822 in the training data set. **(E)** The nomograms for predicting the patients’ OS in the testing data sets. **(F)** The calibration curve for 3-year OS and 5-year OS of the nomogram in the testing data sets. **(G)** The DCA for 3-year OS and 5-year OS of the nomogram in the testing data sets. **(H)** ROC curve analysis showed that AUCs of 3-year and 5-year OS were 0.941 and 0.859 in the testing data set.

We established a nomogram based on the results of Multivariate Cox regression whose indicators include age, gender, neoplasm histologic grade and risk score (**Figure 6A**). The OS of all patients was calculated, and then the 3-year and 5-year OS were predicted. The C-index was calculated to be 0.848 through the “rms” package. The calibration chart (**Figure 6B**) and the clinical decision curve (**Figure 6C**) indicated that the nomogram was of excellent predictive effect. The results of the ROC curve showed that the prediction of the AUC of the 3-year and 5-year OS were 0.882 and 0.822, respectively (**Figure 6D**). Compared with the risk scoring model, the prediction performance of the nomogram has been significantly improved. Similar results were obtained in the validation set (**Figures 6E–H**).

### Enrichment Analysis Identifies Biological Functions and Signaling Pathways Affected by Immune-Related lncRNA in LGG

**Figure 7:**
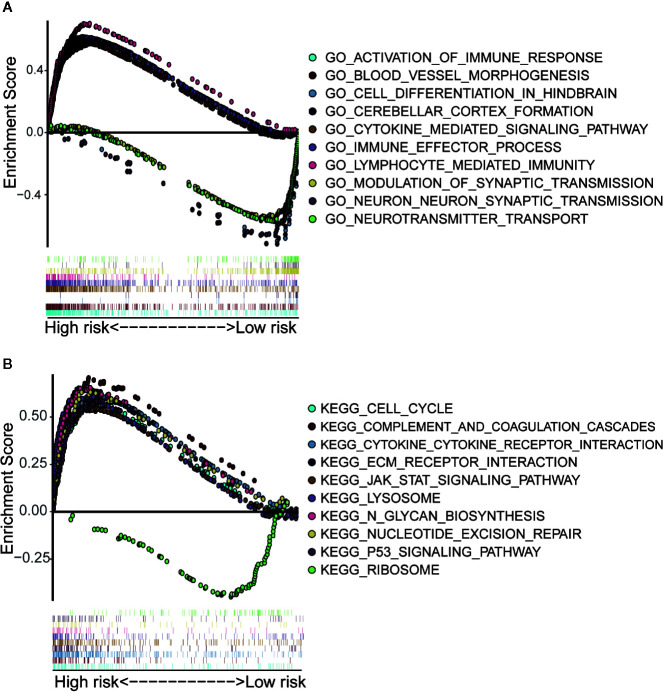
Gene set enrichment analysis of high-risk and low-risk groups. **(A)** GO enrichment analysis indicated that the genes were enriched in several biological processes, such as the activation of immune response, blood vessel morphogenesis, cell differentiation in hindbrain, cerebellar cortex formation, cytokine-mediated signaling pathway, process of immune effect, lymphocyte-mediated immunity, synaptic transmission on neuron, neurotransmitter transport, and the regulation of synaptic transmission function. **(B)** KEGG pathway analysis showed that these genes were involved in the regulation of cell cycle, complementation and coagulation, cytokine-cytokine receptor interaction, ECM receptor interaction, JAK STAT signaling pathway, lysosome, N-glycan biosynthesis, nucleotide excision repair, P53 signaling pathway and ribosome.

We performed GO and KEGG enrichment analysis on the differentially expressed genes of the above high-risk and low-risk groups (**Supplementary Material File 3**). The results of GO enrichment analysis showed that the differential genes mainly played a major role in several biological processes, such as the activation of immune response, blood vessel morphogenesis, cell differentiation in hindbrain, cerebellar cortex formation, cytokine-mediated signaling pathway, process of immune effect, lymphocyte-mediated immunity, synaptic transmission on neuron, neurotransmitter transport, and the regulation of synaptic transmission function (**Figure 7A**). The results of KEGG enrichment analysis showed that these gene sets were involved in cell cycle, complementation and coagulation, cytokine-cytokine receptor interaction, ECM receptor interaction, JAK STAT signaling pathway, lysosome, N-glycan biosynthesis, nucleotide excision repair, P53 signaling pathway, and ribosome (**Figure 7B**). These results are derived from the enrichment of differentially expressed genes in high - and low-risk groups in immune-related lncRNA models. It is helpful for researchers to find possible research directions when studying the mechanism of immune-related lncRNAs in LGG development.

### Correlation Analysis Showed That 11 Immune-Related lncRNAs Were Correlated With Infiltration of Immune Cell Subtypes in LGG

**Figure 8:**
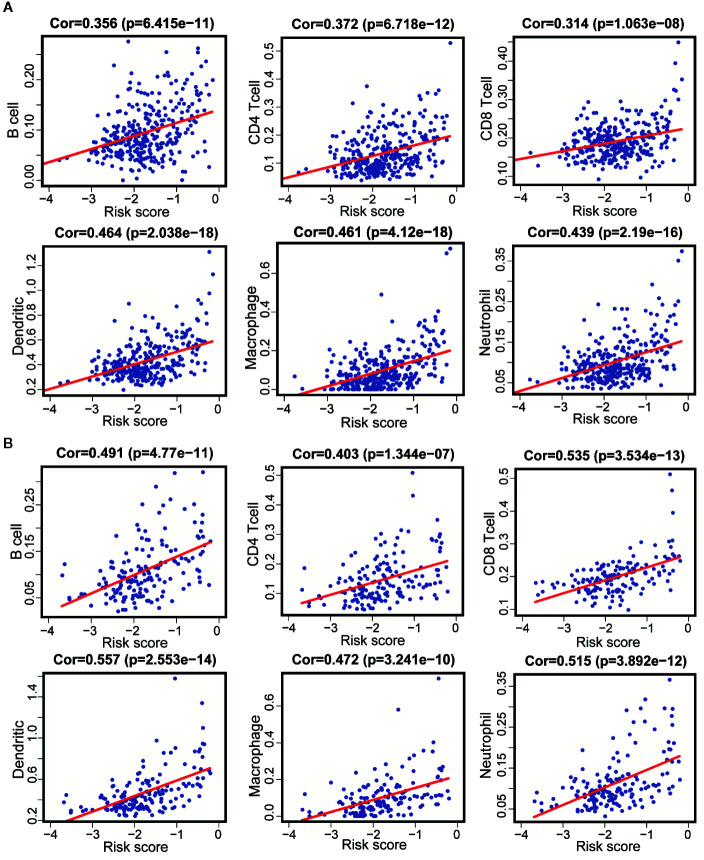
Correlation between 11 immune-related lncRNAs and infiltration of various immune cell subtypes. **(A)** According to the results of the training set that the correlation between the risk scores of B cells, CD4^+^T cells, CD8^+^T cells, DC, neutrophils, and macrophages correlation were 0.356, 0.372, 0.314, 0.464, 0.461, and 0.439 (P < 0.001). **(B)** According to the results of the validation set data that the correlation between the risk scores of B cells, CD4 [+] T cells, CD8 [+] T cells, DC, neutrophils, and macrophages correlation were 0.491, 0.403, 0.535, 0557, 0.515, and 0.472 (P < 0.001).

Since these 11 lncRNAs were immune-related in tumors, we used data from the TIMER database to analyze the correlation between the risk scores of 11 lncRNAs and infiltration of immune cell subtypes in LGG. As shown in **Figure 8A**, the correlation coefficients of risk scores between B cell, CD4^+^T cell, CD8^+^T cell, DC cell, neutrophil and macrophage, and immune-related lncRNA were 0.356, 0.372, 0.314, 0.464, 0.439, and 0.461, respectively (*p* < 0.001). The above results suggested that the infiltration degree of immune cells in tumors was positively correlated with the risk scores of 11 immune lncRNAs. And we got similar results in the verification set (**Figure 8B**).

## Discussion

LGG is composed of astrocytoma and oligodendroglioma, and it is not sensitive to chemotherapy drugs. In recent years, immunotherapy for LGG has become a hot topic (18, 19). It is of great significance to study the relevant factors of tumor prognosis as well as to construct a clinical prognosis model for choosing its treatment for improving the prognosis of patients. Studies by Deng et al. showed that prognostic models based on immune-related genes have been developed and proved to have good predictive performance, which provided theoretical ideas for the immunotherapy of LGG (20). Studies show that many lncNRAs had important effects on the immune microenvironment of gliomas (12), for example, lncRNA H19 could affect the level of immune infiltration of gliomas, as a result of affecting the prognosis of patients (21). In view of the important role of immune-related lncRNAs, Shen et al. applied ssGSEA to identify immune-related lncRNAs associated with the prognosis of breast cancer, and constructed a risk scoring model to predict the OS of patients with breast cancer (14). There is no research yet focusing on constructing a prognostic model of LGG based on ssGSEA to recognize immune-related lncRNAs.

In this paper, we analyzed the expression of lncRNAs in LGG samples in the TCGA database. The LGG patients were divided into high and low immune groups by ssGSEA. Differentially expressed lncRNAs were analyzed between tumor and normal tissues as well as between the high immune group and the low immune group. Through Venn analysis, 36 immune-related lncRNAs were obtained. Univariate Cox analysis and LASSO regression analysis identified 11 key lncRNAs associated with prognosis. Multi-factor Cox analysis was applied to calculate coefficients and construct the risk scoring model. We found that OS of patients in the low-risk group were longer than that in high-risk group. Later we established a nomogram including age, gender, neoplasm histologic grade, and risk scores. By calculating the C-index, drawing the calibration curve, making the clinical decision analysis, and drawing the ROC curve, conclusion is drawn that the nomogram did have good predictive effect. Meanwhile, we obtained similar results in the verification set.

The role of lncRNAs in the immunity of gliomas has been initially explored (10, 22). Among 11 immune-related lncRNAs, AC012558.1, LINC00641, AC126407.1, DGCR9, AC021739.2, FLJ16779, AC124016.2, AL645608.6, AL355916.1, and AC074286 were risk factors for the prognosis of LGG, while AL391422.4 was a protective factor. Previous studies have shown that LINC00461 that serves as competitive endogenous RNA of microRNA-942 could affect the survival of patients with renal cell carcinoma (23). As for breast cancer, the expression level of LINC00641 was negatively correlated with the size of tumor, lymph node metastasis, and clinical stage, which could restrain the proliferation and migration of breast cancer cells (24). lncRNA DGCR9 was up-regulated in gastric cancer tissues, which played the role of promoting cell proliferation, migration and glucose metabolism, and of great importance for the progress of gastric cancer (25). However, the role of above immune-related lncRNAs in the development of LGG still needs further exploration.

In order to explore the potential impacts of these 11 immune-related lncRNAs on cellular immunity of LGG, we analyzed the correlation between the risk score of immune-related lcnRNAs and the infiltration of immune cell subtypes in LGG tissues, which used the data downloaded from the TIMER database. The results showed that the infiltration of B cell, CD4^+^T cell, CD8^+^T cell, dendritic, macrophage, and neutrophil were all positively correlated with risk scores, which confirmed the relatively poor prognosis of patients with high-immunity of LGG in our model. We also got similar results in the verification set. In brief, the 11 lncRNAs we screened were indeed related to the infiltration of immune cells in LGG, which played an important role in predicting the prognosis of patients.

Finally, we performed gene function enrichment analysis to explore the potential roles and functions of these 11 lncRNAs in the development of LGG. The results showed that these lncRNAs were involved in biological processes such as the activation of immune responses, cytokine-mediated signaling pathways, and the regulation of synaptic transmission. At the same time, these lncRNAs played a key role in the regulation of cell cycle, JAK-STAT signaling pathway, repairment of nucleotide excision, as well as P53 signaling pathway. Wang et al. obtained that LncRNA-135528 up-regulates the expression of CXCL10 through JAK/STAT signaling pathway, thereby inhibiting the development of glioma (26). Dp44mT (an Iron Chelator) could restrain the progress of glioma by targeting RORA-mediated activation of ndrg2-IL-6/JAK2/STAT3 signaling pathway (27). The repair of DNA damage could change the M2 polarization of microglial cells and affect the immune microenvironment of glioma through the p53 signaling pathway (28). In short, our results suggested that 11 immune-related lncRNAs had a significant impact on the development of gliomas. However, their specific mechanisms in LGG still need to be further elucidated in *in vitro* experiments.

Here remain several limitations. On the one hand, it was a retrospective study based on a public database without detailed information such as surgical details and clinical treatments. On the other hand, before clinical application, the prediction model constructed in this paper requires more samples for in-depth verification.

## Conclusion

In conclusion, the 11 immune lncRNAs we identified can be used as prognostic markers for patients with LGG. The prognosis prediction performance of the nomogram constructed according to these lncRNAs is relatively effective. In addition, these 11 immune lncRNAs are related to the infiltration of immune cell subtypes in tumor tissues, which play a potential role in the development of LGG through various pathways.

## Data Availability Statement

The data that support the findings of this study are openly available in The Cancer Genome Atlas (TCGA) program at https://portal.gdc.cancer.gov/.

## Author Contributions

JW, YG, and WL designed the research study and analyzed the data from public database. JW, XJ, and WL were involved in data analysis. JW, YW, and LL were responsible for writing of manuscript. XJ, LP, CC, and YG contributed to the revised manuscript. All authors contributed to the article and approved the submitted version.

## Funding

Senior Investigator Research Program of Xiang’an Hospital of Xiamen University (PM201809170016).

## Conflict of Interest

The authors declare that the research was conducted in the absence of any commercial or financial relationships that could be construed as a potential conflict of interest.
